# Genetic testing and gene therapy in retinal diseases: Knowledge and perceptions of optometrists in Australia and New Zealand

**DOI:** 10.1111/cge.14415

**Published:** 2023-08-08

**Authors:** Alexis Ceecee Britten‐Jones, Heather G. Mack, Andrea L. Vincent, Lisa J. Hill, Thomas L. Edwards, Lauren N. Ayton

**Affiliations:** ^1^ Faculty of Medicine, Dentistry and Health Sciences, Department of Optometry and Vision Sciences University of Melbourne Parkville Victoria Australia; ^2^ Faculty of Medicine, Dentistry and Health Sciences, Department of Surgery (Ophthalmology) University of Melbourne Parkville Victoria Australia; ^3^ Centre for Eye Research Australia Royal Victorian Eye and Ear Hospital Melbourne Victoria Australia; ^4^ Eye Department, Greenlane Clinical Centre Auckland District Health Board Auckland New Zealand; ^5^ Faculty of Medical and Health Sciences, Department of Ophthalmology, New Zealand National Eye Centre The University of Auckland Auckland New Zealand; ^6^ School of Biomedical Sciences, Institute of Clinical Sciences University of Birmingham Birmingham UK

**Keywords:** eye care, gene therapy, genetic testing, inherited retinal diseases, ocular genetics, optometry, primary care, retinitis pigmentosa

## Abstract

With advances in gene‐based therapies for heritable retinal diseases, primary eye care clinicians should be informed on ocular genetics topics. This cross‐sectional survey evaluated knowledge, attitudes, and concerns regarding genetic testing and gene therapy for retinal diseases among optometrists in Australia and New Zealand. Survey data included practitioner background, attitudes and practices towards genetic testing for monogenic inherited retinal disease (IRDs) and age‐related macular degeneration, and knowledge of ocular genetics and gene therapy. Responses were received from 516 optometrists between 1 April and 31 December 2022. Key perceived barriers to accessing genetic testing were lack of clarity on referral pathways (81%), cost (65%), and lack of treatment options if a genetic cause is identified (50%). Almost all respondents (98%) believed that ophthalmologists should initiate genetic testing for IRDs and fewer understood the role of genetic counsellors and clinical geneticists. This study found that optometrists in Australia and New Zealand have a high level of interest in ocular genetics topics. However, knowledge gaps include referral pathways and awareness of genetic testing and gene therapy outcomes. Addressing perceived barriers to access and promoting sharing of knowledge between interdisciplinary networks can set the foundation for genetic education agendas in primary eye care.

## Graphical abstract

This survey of 516 optometrists in Australia and New Zealand describes the knowledge, attitudes, and concerns regarding genetic testing and gene therapy for retinal diseases among primary eye care practitioners in this region.

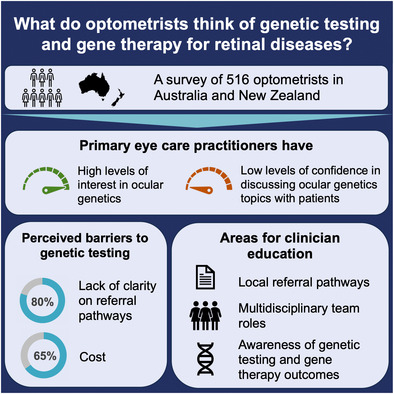

## 1 INTRODUCTION

Genetic medicine is a rapidly evolving field, and sensitive and specific genetic tests now exist for monogenic inherited retinal diseases (IRDs) caused by variants in single genes. IRDs affect approximately 1 in 4000 individuals and, to date, over 300 genes have been identified to cause IRDs.^1^ Common monogenic IRD phenotypes include photoreceptor diseases, such as retinitis pigmentosa and cone and cone rod dystrophies; macular dystrophies, such as Stargardt disease and Best disease; and many others. There is significant clinical and genetic heterogeneity between IRDs; variants in a single gene can cause different IRD phenotypes inherited in various inheritance patterns, and each phenotype can be caused by variants in different genes.^2^, ^3^ For monogenic IRDs, genetic testing is an important strategy to confirm diagnosis and allows informed genetic counselling, particularly for family planning purposes.^4^ A confirmed genetic diagnosis is also a minimum requirement in determining an individual's eligibility for gene‐specific treatments. In 2017, Luxturna® (voretigene neparvovec‐rzyl) became the first ocular gene treatment approved internationally.

Gene‐specific treatments are being developed for not only IRDs,^5^ but also more common retinal diseases like age‐related macular degeneration (AMD).^6^ In contrast to IRDs, AMD is a complex heritable disorder caused by the interactions of changes in multiple genes and environmental factors. In AMD, the presence of any one of the disease‐associated variants is not entirely responsible for disease development.^4^

Diagnostic genetic testing is recommended for all individuals with presumed single‐gene IRDs.^4^, ^7^ However, for complex disorders such as AMD, routine genetic testing is not currently available or recommended.^4^ Nevertheless, genetic testing is useful for selecting suitable candidates for clinical trials of novel gene‐based therapies^8^; for example, the efficacy of gene therapy for AMD patients with variants in the *CFI* gene.^6^ Therefore, it is vital that clinicians are aware of the evidence supporting diagnostic testing strategies and their implications and potential outcomes.

The provision of genetic testing entails not only ordering a genetic test but also interpreting the result in the context of the clinical findings and patient counselling.^4^ Pre‐testing counselling is important for ensuring that patients are prepared for the implications of the results.^9^ The uptake of IRD genetic testing in Australian private ophthalmology clinics is currently estimated to be around 10%, reflecting historic management patterns and access to genetic services.^10^ Currently, the likelihood that a genetic test will provide an IRD diagnosis is approximately 60%.^11^ However, with the increasing availability of sponsored genetic testing programs,^12^, ^13^, ^14^ it's likely that diagnostic genetic testing will be available to more patients with retinal diseases. Thus, there is an increasing need to enhance the genomics literacy of eye care professionals to meet patient needs,^15^ manage expectations,^16^ and support the integration of multidisciplinary care models.^17^, ^18^

Optometrists are usually patients' first point‐of‐contact in the healthcare system and play an essential role in identifying patients with retinal diseases, coordinating co‐management, and providing long‐term care.^19^, ^20^ Australia and New Zealand have a uniform approach for the standards of optometry training, with continuing professional development activity required to maintain licensure.^21^ Optometric scope of practice includes independent diagnosis and management of ocular disorders relating to visual function and eye health.^21^, ^22^ Optometrists who hold therapeutic medicine endorsement can prescribe topical, and in New Zealand oral, medications for managing eye diseases.^22^ It is essential for optometrists to stay updated with the latest evidence in diagnosing heritable retinal diseases and genetic testing strategies so that patients are appropriately managed and informed of emerging treatments. Few studies have evaluated genetics and gene therapy knowledge among eyecare clinicians. A study conducted between 2009 and 2012 found broadly supportive attitude to genomic medicine among eye care professionals in the UK,^23^ and a 2019 survey found a low level of understanding of IRD genetics and genetic testing availability amongst US‐based optometrists and ophthalmologists.^24^ Both studies had fewer than 40 optometrist respondents.

The aim of this study was to evaluate knowledge, attitudes, and concerns towards genetic testing and gene therapy for retinal diseases among optometrists in Australia and New Zealand.

## 2 METHODS

The project was approved by the Royal Australian and New Zealand College of Ophthalmologists (RANZCO) Human Research Ethics Committee (Reference: 135.22). All participants have informed consent prior to undertaking the survey.

### 2.1 Survey development

Survey questions were developed based on published genetics questionnaires amongst health care practitioners,^23^, ^25^, ^26^ and modified for retinal diseases in consultation with a multidisciplinary team (optometrists and ophthalmologists). The survey comprised 31 questions (Table 1). The questions within each section required responses that include: Yes/No, select all that apply, select one option only, and rating on 3‐ or 5‐point Likert scales.

**TABLE 1:** Summary of survey questions.

Survey topic	Topic
Practitioner demographics and practice modality	Years in clinical practice Country and region Country of optometry clinical training Primary clinical setting Principal type of work Age and gender Therapeutic endorsement Hours typically spent providing patient care (in a clinical setting)
Practices relating to ocular genetics	Frequency of seeing patients with AMD in clinical practice Frequency of seeing patients with IRD in clinical practice? (Examples given as retinal dystrophy, macular dystrophy, congenital stationary night blindness and was specified to exclude AMD) Confidence in discussing topics relating to genetic testing and gene therapy to patients
Attitudes towards genetics testing for retinal diseases	Whether they have ever recommended to patients to have a genetic test for their retinal disease Perception on which profession(s) should play a role in initiating genetic testing for patients with retinal diseases Perceived barriers to genetic testing for patients with retinal diseases
Perception of ocular genetics and gene therapy	Level of agreement/disagreement with the following statements: Genetic testing for eye diseases is as important as that for cancers Diagnostic genetic testing should now be routine practice for people with rare inherited retinal diseases, like rod‐cone dystrophy, as well as more common conditions, like AMD I would not recommend genetic testing for eye diseases where no treatment is available If I personally had a genetic eye disease, I would have genetic testing if it was available to me If I personally had a genetic eye disease, I would undergo gene therapy if it was available to me Gene therapy is or will soon become a useful treatment strategy for rare retinal diseases, such as rod‐cone dystrophy/retinitis pigmentosa Gene therapy is or will soon become a useful treatment strategy for more common retinal diseases, such as age‐related macular degeneration Level of concerns about factors relating to gene therapy for retinal diseases
Sources of information	Source(s) of information and/or evidence used to learn about genetic testing and gene therapy for retinal diseases
Knowledge of ocular genetics and gene therapy topics (and scored out of 9. Answers in Table S1)	Knowledge of ocular genetics Knowledge of which conditions are monogenic diseases (disease traits are controlled by a single gene) The carrier of a disease gene may be completely healthy Using current technology, approximately one in three ocular genetic tests may come back with an inconclusive result Gene therapy and stem cell therapy are the same treatment Patients in Australia have now been treated with approved ocular gene therapy products The main goal of gene therapy for the eye is to slow down the disease Having gene therapy for their eye condition means a person will not pass on an eye condition to any children they may have in the future

Abbreviation: AMD, age‐related macular degeneration.

Sections one to five assessed practitioners' demographics, as well as their perception, attitudes, and practice patterns relating to genetic testing and gene therapy. Section six evaluated practitioners' knowledge of ocular genetics and gene therapy concepts using questions based on the Attitudes to Gene Therapy–Eye tool.^27^, ^28^, ^29^ Their knowledge was quantified to give an overall knowledge score out of nine. A point was given to each correct answer in the knowledge section, with zero representing all answers incorrect and nine all answers correct (Table S1).

### 2.2 Data collection

Data were collected between 1 April and 31 December 2022 and administered using Qualtrics (Provo, UT, USA). The survey was distributed to optometrists in Australia and New Zealand through newsletters of professional organisations and networks (Optometry Australia, Specsavers, OPSM, Australian College of Optometry, New Zealand Optics). Additionally, 1600 postal invitations were sent to practitioners in Australia randomly selected from the Optometry Australia “Find an Optometrist” public portal (searched 1 April 2022). Practitioners who received the mail out invitation could either complete the survey online or a hard‐copy. As data collected were non‐identifiable, reminders were not sent.

### 2.3 Data analysis

Sample size was calculated based on 7000 registered optometrists in Australia and New Zealand. Based on 95% confidence level and 2% margin of error, the sample size was 360 participants.

Statistical analysis was performed using R for statistical consulting (v4.1.2; R Core Team 2021). Responses with >80% completion rate (i.e., all sections complete up to knowledge) were included in the analysis. Responses between practitioners in Australia and New Zealand were compared using the Fisher exact test and if they were similar, data from both regions were pooled. Descriptive methods were used, summarising the frequency and percentages of responses for categorical measures. Continuous variables were summarised as median (SD) for parametric or median (IQR) for non‐parametric distribution.

Univariate and multivariate binocular logistic regression analyses was performed to assess factors associated with whether optometrists (i) have recommended genetic testing for people with retinal diseases and (ii) think that optometrists should play a role in initiating genetic testing for retinal disease. Independent variables included age, gender, country, region, practice setting, years in practice, hours worked, knowledge of ocular genetics, if they would personally have genetic testing if they had an IRD, and confidence in discussing different topics relating to genetic testing. Variables significantly associated with the outcome were evaluated in the multivariable model. A *p*‐value of <0.05 was considered statistically significant.

## 3 RESULTS

Of 541 responses received, 516 were included in the analysis (including 498 completed responses and 18 with all questions answered except for knowledge). The sample represented 8% of registered optometrists in Australia and 4% of registered New Zealand optometrists. Approximately 45% of the responses were recorded online (231 online responses; 285 paper responses).

Table 2 summarises the demographics of participating optometrists. Most respondents completed their optometry training in Australia or New Zealand (94%), and most currently practice in Australia (95%). Half of the respondents (50%) primarily practice in metropolitan settings, 39% in regional or suburban settings, and 11% in rural settings. The highest representation was of practitioners in independent practices (59%), followed by corporate practices (33%). Three of four respondents (76%) had therapeutic endorsement. There were no significant differences in the distribution of responses from optometrists in Australia and New Zealand, and responses were thereafter pooled.

**TABLE 2:** Participant characteristics.

Participant characteristic (*n* = 516)	Number and percentage (%) of responses unless otherwise indicated
**Age**, median (IQR), years	43 (30–55)
**Gender**	
Male	227 (44%)
Female	285 (55.2%)
Different term/prefer not to say	4 (0.8%)
**Therapeutic endorsement**	
Yes	393 (76.2%)
No	123 (23.8%)
**Country**	
Australia	488 (94.6%)
New Zealand	28 (5.4%)
**Setting**	
Metropolitan	256 (49.6%)
Regional/Suburban	203 (39.3%)
Rural	57 (11%)
**Training**	
Australia or New Zealand	487 (94.4%)
Other	29 (5.6%)
**Years in optometry practice, median (IQR)**	19 (7–31)
**Number of hours in clinical practice per week,** median (IQR)	35 (25–40)
**Principal practice setting**	
Academic or research	16 (3.1%)
Corporate practice	169 (32.8%)
Hospital or public health clinic	16 (3.1%)
Independent practice	304 (58.9%)
Refractive surgery clinic	3 (0.6%)
Other	8 (1.6%)

Abbreviation: IQR, interquartile range.

Most optometrists reported that they would see patients with AMD in their clinical practice every week or more (56%); 25% would see 1–3 per month, 12% would see 1 per 1–2 month, and 7% would see fewer than one every 6 months. For IRDs, 44% of optometrists would see fewer than 2 cases per year, 36% see a case every 2–6 months, 14% see a case every 1–2 months, and 6% see an IRD patient every month or more.

**FIGURE 1:**
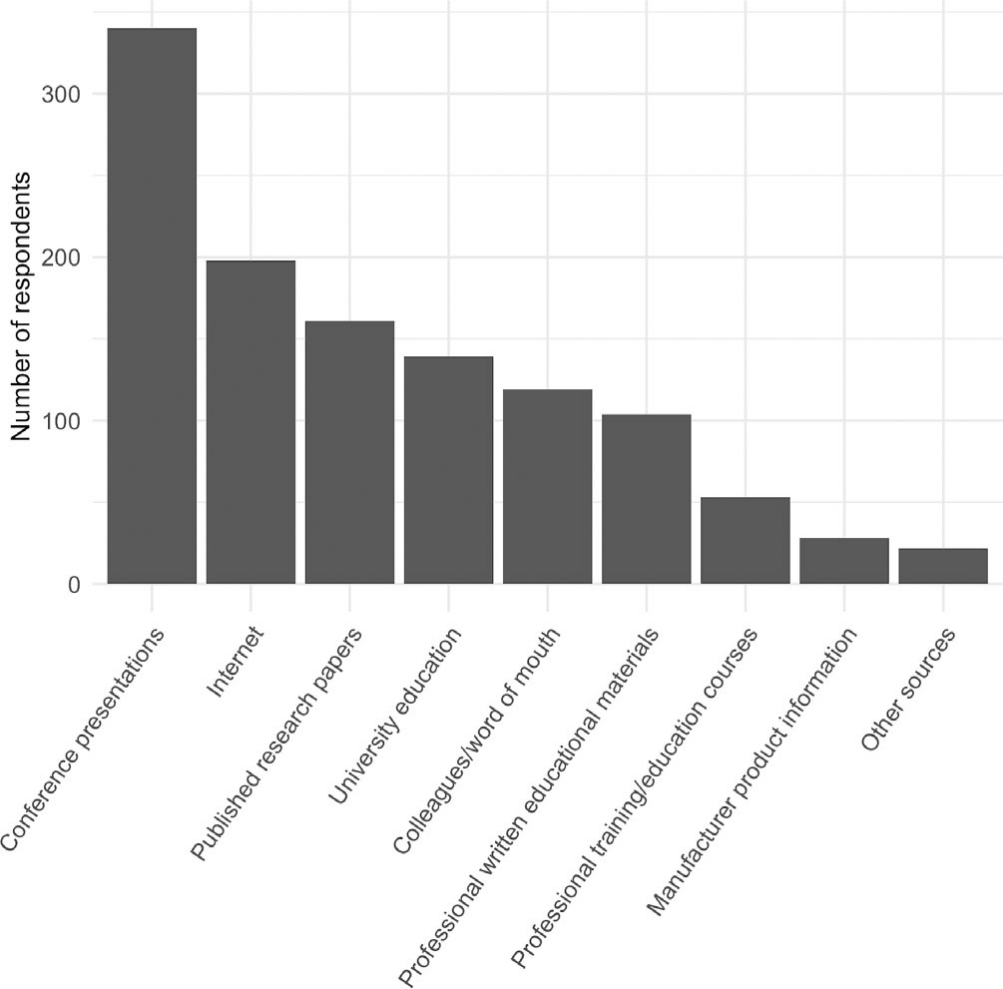
Sources of information and/or evidence optometrists indicated to have used to learn about genetic testing and gene therapy for retinal diseases.

The major sources of information optometrists indicated they have used to learn about genetic testing and gene therapy were conference presentations (68% of respondents) and internet (40% of respondents). A third of optometrists (32%) indicated using published primary research papers to learn about genetic testing and gene therapy (Figure 1).

### 3.1 Attitudes and perceptions of genetic testing and gene therapy for retinal diseases

**FIGURE 2:**
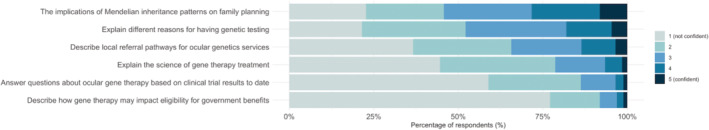
Optometrists self‐reported confidence in discussing ocular genetics topics with a patient. Responses are indicated as: (1) I would not be able to answer questions on this topic; (2) I have some knowledge but would not feel comfortable answering questions on this topic; (3) I could answer a few basic questions about this topic; (4) I feel comfortable answering questions about this topic; (5) I feel confident answering questions on this topic. [Colour figure can be viewed at wileyonlinelibrary.com]

Most respondents (over 72%) indicated that they could, at most, answer a few basic questions about genetic testing and ocular gene therapy (Figure 2). Optometrists generally indicated higher levels of confidence in discussing topics related to implications of genetic testing (Mendelian inheritance patterns, reasons for having genetic testing, and referral pathways) than gene therapy (science of gene therapy, clinical trial results to date, and the impact of treatment on eligibility for government benefits).

**FIGURE 3:**
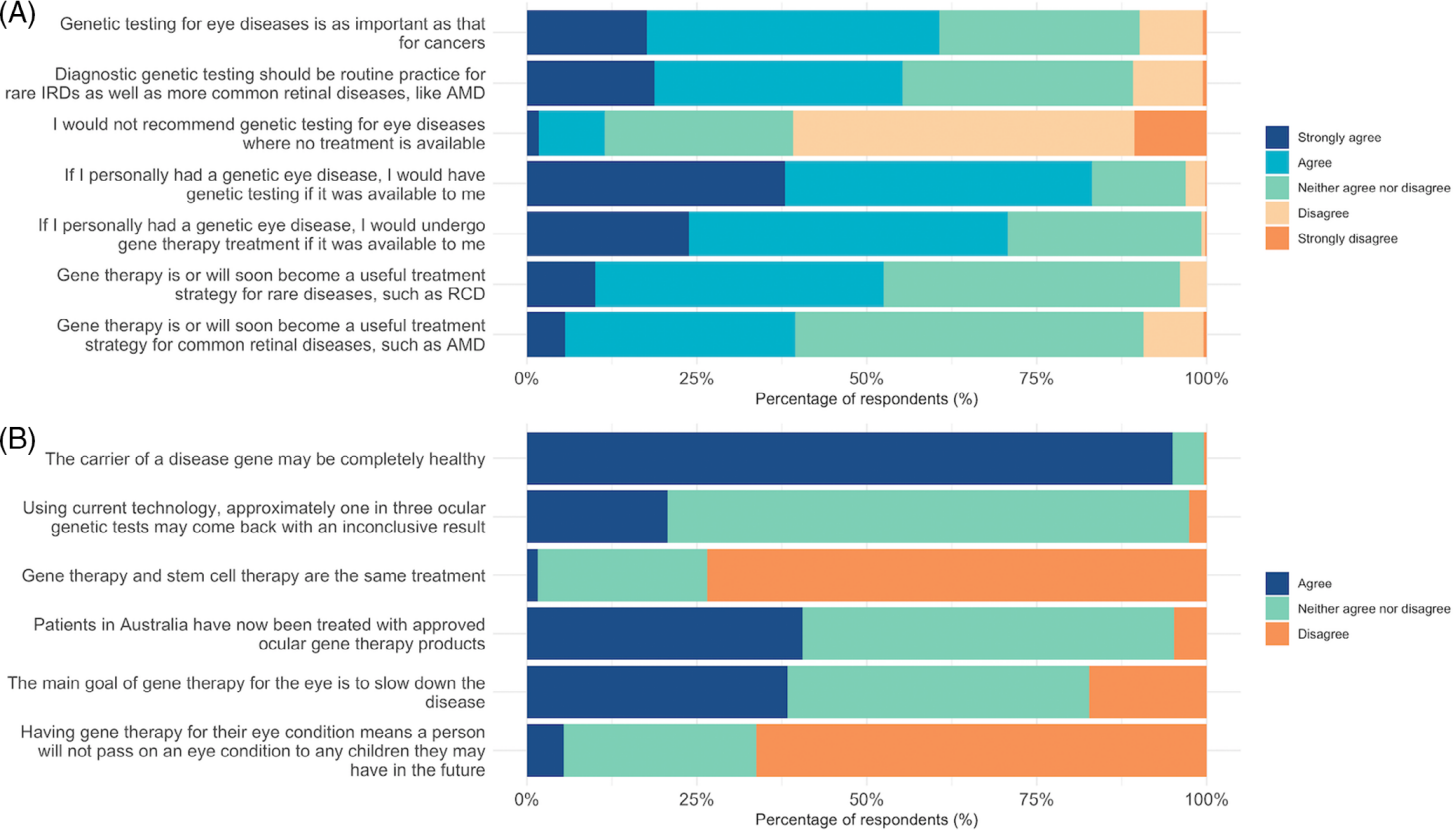
Level of agreement with statements relating to (A) perceptions of ocular genetics and gene therapy and (B) knowledge of genetic testing and gene therapy concepts, from *n* = 516 optometrists. AMD, age‐related macular degeneration; RCD, rod‐cone dystrophy. [Colour figure can be viewed at wileyonlinelibrary.com]

Of the 516 respondents, 61% agreed or strongly agreed with the statement that genetic testing for eye diseases is as important as that for cancers, and 55% agreed or strongly agreed that diagnostic genetic testing should be routine practice for both IRDs and AMD (Figure 3A). Only 11% of respondents indicated that they would not recommend genetic testing for eye disease where no treatment is available, whereas 61% disagreed with that statement.

On whether gene therapy would soon become a useful treatment strategy for retinal disease, 52% of respondents agreed/strongly agreed that it will be for rare diseases, such as rod‐cone dystrophies, but only 39% indicated the same for common retinal disease, such as AMD. Nonetheless, if practitioners personally had a genetic eye disease, 83% of respondents indicated that they would have genetic testing, and 71% would have gene therapy, if these were available to them.

**FIGURE 4:**
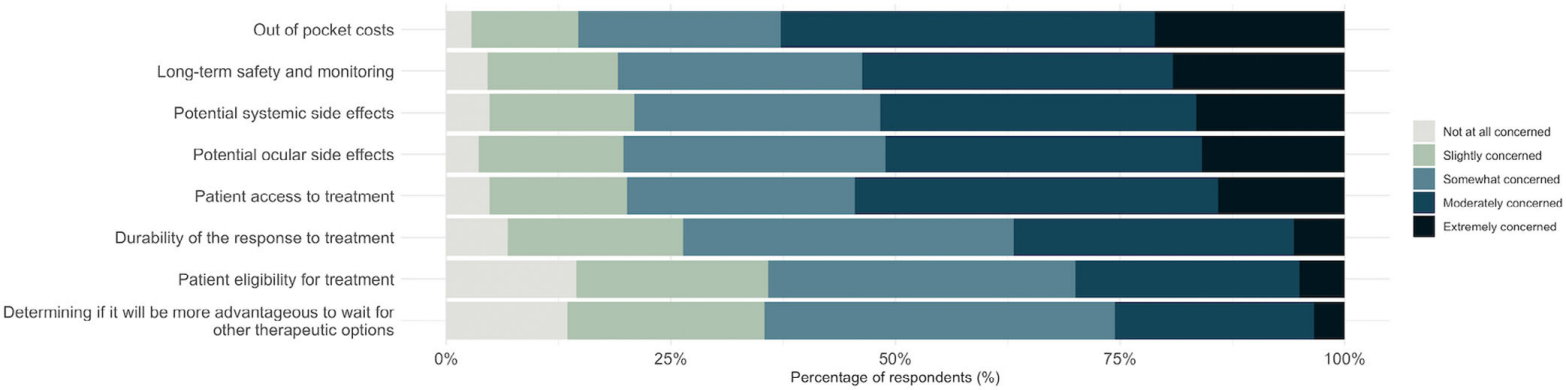
Optometrists' level of concern about factors relating to gene therapy for retinal diseases. [Colour figure can be viewed at wileyonlinelibrary.com]

Regarding concerns towards gene therapy for retinal diseases (Figure 4), key concerns, each indicated by over 50% of respondents to be of moderate or extreme concern, are out of pocket costs (63% responses), patient access to treatment (55% responses), long‐term safety and monitoring (53% responses), and potential systemic (52% responses) and ocular (51% responses) side effects.

### 3.2 Practices relating to genetic testing for retinal diseases

Of the 516 optometrists, 37% have previously recommended for patients to have a genetic test for their retinal disease. Univariate and multivariate binary logistic regression analysis found that participants' practice region and confidence in discussing genetic testing topics were associated with whether they have recommended genetic testing to patients with retinal diseases (Table S2). Optometrists in rural areas were twice more likely to have recommended genetic testing than those in metropolitan regions (OR = 1.97 [95%CI: 1.02–3.81]). Optometrists who felt comfortable discussing reasons for getting genetic testing were 3–4 times more likely to have recommended genetic testing than those who had little/no confidence in discussing these topics (some confidence: OR = 2.85 [95%CI: 1.72–4.80]. Comfortable/confident: OR = 3.74 [95%CI: 1.95–7.26]). Similarly, practitioners who felt comfortable describing local ocular genetics referral pathways were 3–5 times likely to have recommended patients to have genetic testing for retinal diseases than those who had no/little confidence discussing these topics (some confidence: OR = 2.92 [95%CI: 1.82–4.72]. Comfortable/confident: OR = 4.96 [95%CI: 2.75–9.18]).

**FIGURE 5:**
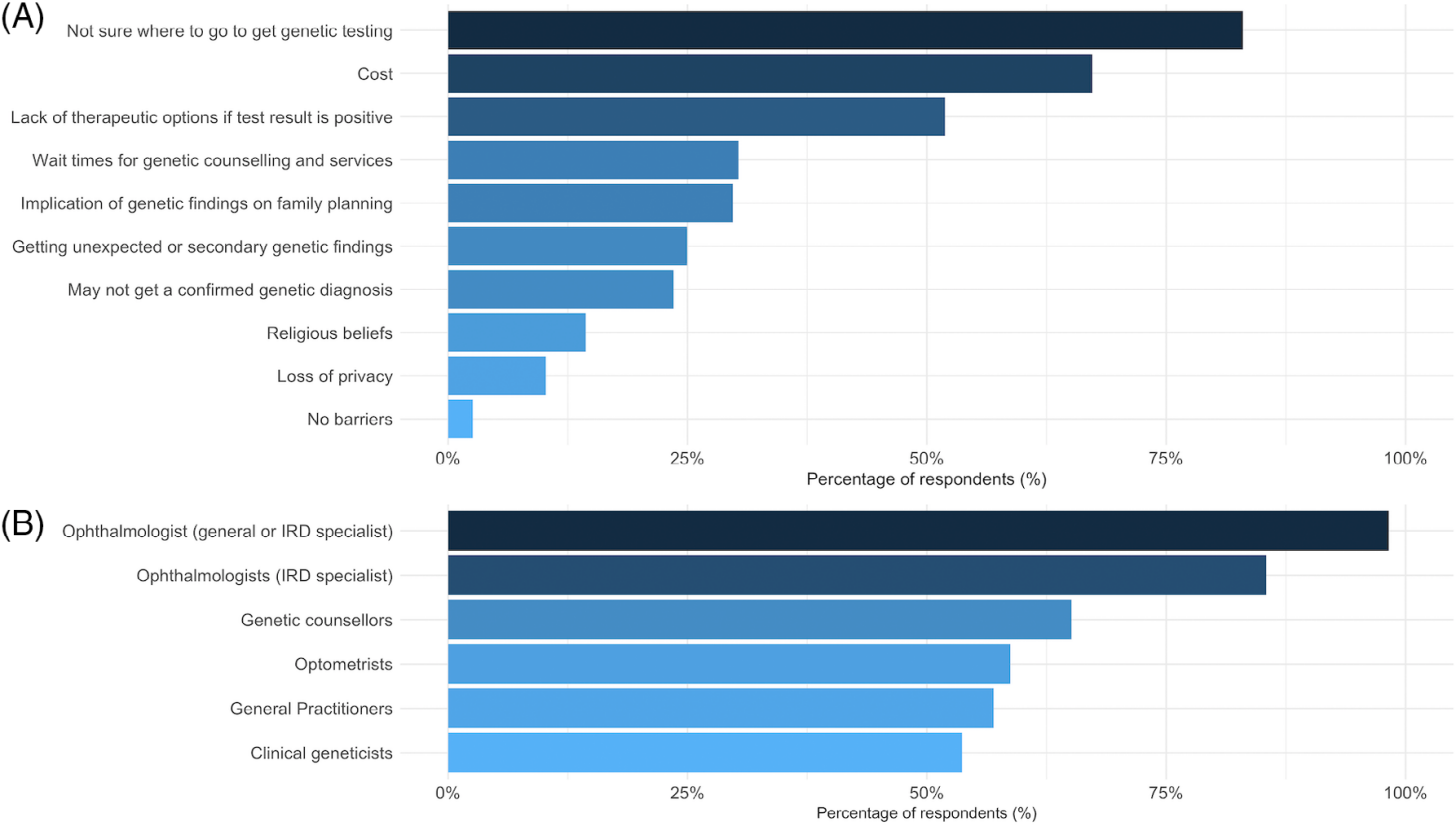
Perceived barriers to genetic testing and multidisciplinary roles. Percentage of responses from 516 optometrists on (A) factors they consider to be a significant barrier to getting genetic testing for retinal diseases and (B) professions they think should play a role in initiating genetic testing for patients with retinal disease. IRD, inherited retinal disease. [Colour figure can be viewed at wileyonlinelibrary.com]

Figure 5A shows factors that optometrists consider to be significant barriers to getting genetic testing for retinal diseases. The key barriers, each selected by over 50% of respondents, were not sure where to go to get genetic testing (81% of respondents), cost (65% of respondents), and lack of therapeutic options if test result is positive (50% of respondents).

Regarding multidisciplinary team roles in ocular genetics patient care (Figure 5B), almost all the respondents (98%) indicated that ophthalmologists (general or subspeciality‐trained IRD specialists) should play a role in initiating genetic testing for patients with retinal disease. Of these, 74% selected both general and IRD specialists and 13% selected only sub‐speciality trained IRD ophthalmologists. Approximately 60% of optometrists thought that optometrists could also play a role in initiating genetic testing for retinal diseases. A similar number acknowledged the role of non‐ophthalmic clinicians in initiating genetic testing for patients with retinal diseases, with 65% selecting genetic counsellors, 54% selecting clinical geneticists, and 57% selecting general practitioners.

Optometrists' perception of whether they think they should play a role in initiating genetic testing for patients with retinal diseases was associated with practitioner's age, whether they would personally have genetic testing if they had an IRD, and their confidence in discussing genetic testing topics (Table S3). Optometrists aged <30 were more likely to agree that they should initiate genetic testing for patients with retinal diseases than practitioners aged 30–45 (OR = 1.89 [95%CI: 1.12–3.23]). Optometrists who would personally have genetic testing if they had a genetic eye disease were twice as likely to think that they should initiate genetic testing for patients with retinal diseases than those who would not (OR = 2.39 [95%CI: 1.45–3.85]). Optometrists who felt comfortable explaining different reasons for having genetic testing were up to three times more likely to agree than those who had little/no confidence discussing this topic (some confidence: OR = 1.67 [95% CI: 1.04–2.70]. Comfortable/confident: OR = 3.01 [95%CI: 1.54–6.05]).

### 3.3 Knowledge of ocular genetics and gene therapy concepts

Regarding optometrists' knowledge of genetic testing and gene therapy for retinal diseases (Figure 3B; Table S1), the overall median knowledge score was 6 (IQR: 5–7) out of 9. Most practitioners knew that the carrier of a disease gene may be completely healthy (95% correct). Most respondents correctly indicated that gene therapy and stem therapy are not the same treatment (74% correct) and having gene therapy does not mean that a person will not pass an eye condition to any children they may have in the future (66% correct). Fewer respondents knew that patients in Australia have now been treated with approved ocular gene therapy products (41% correct), the main goal of gene therapy for the eye is to slow down the disease (38% correct), and approximately 1 in 3 ocular genetic tests may return an inconclusive result (21% correct).

## 4 DISCUSSION

This study describes optometrists' attitudes and perceptions towards, as well as knowledge about, genetic testing and gene therapy for retinal diseases. To our knowledge, this is the first study of this kind to evaluate self‐reported ocular genetics practices among primary eye care practitioners in Australia and New Zealand, with responses from 516 optometrists (approximately 8% of all registered practitioners).

We report a high level of interest towards ocular genetics topics amongst optometrists in primary eye care settings. In contrast to a 2020 study that found 11% of US‐based optometrists (based on *n* = 36 respondents) have ordered genetic tests and 25% have referred a patient to a genetic counsellor,^24^ 37% of optometrists from the present study have recommended for patients to have a genetic test for their retinal disease. Practitioners in rural areas and those who felt more comfortable discussing genetic testing reasons and referral pathways were more likely to have made this recommendation. Optometrists identified key barriers to genetic testing as cost, access, and lack of therapeutic options if a genetic cause is identified. Consistent with the literature, clinicians' decision to offer genetic testing is influenced by environmental context and resources and beliefs about consequences.^25^ Thus, raising clinician awareness about the values of genetic tests and implications on patient outcomes, as well as addressing structural constraints of access, may set the foundation for addressing barriers to genetic testing.

We also report modest knowledge regarding ocular genetics and gene therapy among optometrists, with a median knowledge score of 6 out of 9. Several studies have identified deficiencies in genetics and genetic testing knowledge amongst various areas of healthcare practice,^25^, ^26^, ^30^, ^31^ but only one other study included primary eye care practitioners. This study by Ganne et al. (conducted between 2009 and 2012) found a relatively low level of ocular genetics knowledge among 35 UK‐based optometrists, dispensing optometrists, and optometry clinic staff.^23^ Differences in knowledge scope may arise from differences in practice scope or an increased ocular genomics literacy amongst eye care professionals following the approval of the world‐first ocular gene therapy treatment in 2017. Compared to patients with IRDs,^27^ optometrists had better knowledge of gene therapy methods and awareness of potential outcomes. Notably, while 60% of respondents suggested that optometrists could play a role in initiating genetic testing for patients, self‐reported confidences in discussing ocular genetics concepts with patients were generally low. Most respondents indicated that they did not feel comfortable answering questions on genetic testing topics, including implications of Mendelian inheritance patterns on family planning and different reasons for having genetic testing. To support a potential role for optometrists in initiating discussions about genetic testing, further training is needed in both ocular genetics (e.g., implications of genetic testing for different heritable eye diseases and ocular gene therapy research) and foundational genetics (e.g., types of genetic tests and inheritance patterns).

Upskilling primary eye care practitioners on ocular genetics concepts can significantly improve genetics care provision and strengthen integrated ocular genetics networks. Improved health literacy amongst primary eye care practitioners could help improve equity of demand through a shared decision‐making process and improve testing efficiency.^32^, ^33^ Additionally, knowledge of genetics associated with different IRDs can assist with patient management and more accurate referrals to specialist care. For example, syndromic IRDs are associated with systemic manifestations, such as kidney and heart disease.^34^ Refsum disease can be managed with a low phytanic acid diet,^35^ so an accurate genetic diagnosis is vital for informing clinical management.

Generally, genetic testing is initiated by IRD specialists and geneticists at large academic research centres.^4^ Potential barriers to access in this model include not enough ocular genetics specialists to meet rising demand for genetic testing and provide adequate counselling, and individuals from geographically isolated centres who are unable to travel.^9^ Within interdisciplinary networks, optometrists in primary eye care could support IRD and ocular genetic specialists by identifying patients with IRDs and updating them on emerging research evidence and therapies. In rural communities without easy access to ocular genetics services, optometrists could contribute to multidisciplinary ocular genetics teams by providing clinical information, such as retinal imaging data, and assisting with virtual consults. In research settings, optometrists are also involved in screening patients for susceptibility genes to assess their eligibility for clinical trials.^36^, ^37^

Our findings highlighted several knowledge gaps in primary eye care, including awareness of genetic testing outcomes, as only 20% of optometrists knew that approximately 1 in 3 ocular genetic tests may return an inconclusive result; practical considerations (cost, ways to access, impact on health and medical insurance); and knowledge of gene therapy clinical trial results to date. In this study, we considered 1 in 3 ocular genetic tests being inconclusive as the correct answer to reflect the approximate number of cases in whom a candidate pathogenic variant can be identified as being causative of their IRD, based on data from next generation sequencing studies^11^ and Australian IRD registries.^12^, ^38^ However, in practice, the interpretation of genetic testing results is complex, often requiring additional testing (e.g., to determine phase or to evaluate additional variants) and multidisciplinary team input. The diagnostic rate also varies depending on the testing strategy and criteria used to assign pathogenicity. Notwithstanding these considerations, this data intends to demonstrate the importance for clinicians to acknowledge the limitations of current genetic testing sequencings during pre‐test counselling. Other key information needs previously indicated by patients with IRDs include patterns of inheritance, risk to other family members, and the process of genetic testing, and healthcare professionals are the preferred sources for obtaining this information.^15^

Another key area for further education is the role of genetic care providers in delivering patient care for heritable retinal diseases. Both ophthalmic and genetic providers have essential roles in diagnosing IRDs and often provide coordinated services.^39^ Affected individuals need to be appropriately evaluated by an ophthalmologist, preferably an IRD specialist, with the expertise to make a provisional clinical diagnosis.^4^, ^7^ Genetic counsellors and geneticists also play an important role in delivering pre‐ and post‐test genetic counselling, which now often includes implications on emerging therapies. Integration of knowledge and sharing of information, including information regarding services, between primary eye health practitioners and other ocular genetics care providers (ophthalmologists, genetic counsellors) is critical for providing coordinated, patient‐centred care, to optimise health outcomes for people with retinal diseases.^39^

We propose several strategies to assist in the implementation of genomics education in primary eye care. Based on sources of information and/or evidence indicated by optometrists, conference presentations and continuing education courses, such as workshops held in conjunction with professional conferences, are potential approaches for providing genetics education. Both case‐based workshops and blended learning courses (including workshops and interactive online resources) have been shown as effective methods for educating non‐genetics medical specialists to increase their understanding of genomic medicine and its applications.^40^

The goal of education is to provide not only foundational learning to increase practitioners' knowledge and confidence in ocular genetics concepts, but also resources to apply this knowledge to clinical practice.^41^ A key barrier to genetic testing, selected by over 50% of respondents, was “not sure where to go to get genetic testing”. Educational content could be supplemented with practical and updated information on local ocular genetics specialists and referral pathways. Co‐designing and delivering educational content with ophthalmologists, genetics specialists (such as geneticists and genetic counsellors), and other health care providers (such as general practitioners) could assist in the translation and adaptation of evidence appropriate to each specialty and strengthen interdisciplinary relationships.

A key strength of this study is the high level of representation of primary eye care practitioners in the Australasia region. Our cohort (with *n* > 500) exceeded the a priori sample size requirement and represents 8% of all registered optometrists in Australia and New Zealand. Our sample had a similar age and gender distribution, therapeutic endorsement, and geographic distribution with Australia and New Zealand optometry board registration data, showing that our responses can be extrapolated to the population being studied. However, while the cohort represented a broad range of demographics and practice modalities, there was the potential for self‐selection bias, as responses may be predominantly from clinicians with an interest in ophthalmic genetics. We combined responses from New Zealand and Australia as there were no differences in response distributions; however, we recognise there are differences in healthcare structures between the two countries. Our findings are also of practitioners predominantly trained and practising in Australia and New Zealand. Globally, there is substantial variation in the scope of practice of optometrists,^37^, ^42^ and our results may not be generalizable to other countries and practice settings.

## 5 CONCLUSIONS

This study provides new insights into optometrists' interests towards ocular genetics topics and highlights knowledge gaps and perceived barriers to genetic testing. In primary care, optometrists can contribute towards multidisciplinary networks by providing clinical information, coordinating co‐management, and identifying patients for research studies. Potential areas for education in primary eye care include awareness of potential outcomes of genetic testing and gene therapy, access to genetic testing, and sharing of knowledge between genetic and ophthalmic care providers. Empowering optometrists with tailored knowledge on these topics can improve the care of heritable retinal diseases, maximising the potential clinical benefit to affected families.

## AUTHOR CONTRIBUTION STATEMENT

Conceptualization: Alexis Ceecee Britten‐Jones, Lauren N. Ayton; Methodology: Alexis Ceecee Britten‐Jones, Lauren N. Ayton, Heather G. Mack, Andrea L. Vincent, Lisa J. Hill, Thomas L. Edwards; funding acquisition: Alexis Ceecee Britten‐Jones, Lauren N. Ayton, Andrea L. Vincent, Lisa J. Hill, Thomas L. Edwards; data collection: Alexis Ceecee Britten‐Jones; analysis and interpretation of results: Alexis Ceecee Britten‐Jones; Writing – Original Draft: Alexis Ceecee Britten‐Jones; Writing – Review & Editing: Alexis Ceecee Britten‐Jones, Lauren N. Ayton, Heather G. Mack, Andrea L. Vincent, Lisa J. Hill, Thomas L. Edwards. All authors reviewed the results and approved the final version of the manuscript.

## FUNDING INFORMATION

This study was funded by a Universitas 21 Health Sciences Group International Projects Fund (Alexis Ceecee Britten‐Jones, Andrea L. Vincent, Lisa J. Hill, Thomas L. Edwards, Lauren N. Ayton) and University of Melbourne Early Career Researcher Grant (Alexis Ceecee Britten‐Jones). Lauren N. Ayton is supported by a National Health and Medical Research Council Investigator Grant (GNT#1195713) and University of Melbourne Driving Research Momentum Fellowship. The funder has no role in the design and conduct of the study; collection, management, analysis, and interpretation of the data; preparation, review, or approval of the manuscript; or decision to submit the manuscript for publication.

## CONFLICT OF INTEREST STATEMENT

The authors declare no conflicts of interest.

### PEER REVIEW

The peer review history for this article is available at https://www.webofscience.com/api/gateway/wos/peer-review/10.1111/cge.14415.

## ETHICS STATEMENT

The project was approved by the Royal Australian and New Zealand College of Ophthalmologists (RANZCO) Human Research Ethics Committee (Reference: 135.22).

## Supporting information


**Data S1:** Supporting Information.

## Data Availability

The data that support the findings of this study are available from the corresponding author upon reasonable request.
