# Supplementary material for: Genetic testing and gene therapy in retinal diseases: Knowledge and perceptions of optometrists in Australia and New Zealand
Source: Clin Genet. 2023 Aug 8;105(1):34–43. doi: 10.1111/cge.14415 (PMC10952375; doi:10.1111/cge.14415)
Supplement: Supplementary file 1 — Data S1: Supporting Information. [file CGE-105-34-s001.docx]

**Supplementary tables:** Genetic testing and gene therapy in retinal diseases: Knowledge and perceptions of optometrists in Australia and New Zealand

**Authors**: Alexis Ceecee Britten-Jones, Heather G. Mack, Andrea L. Vincent, Lisa J. Hill, Thomas L. Edwards, Lauren N. Ayton.

**Supplemental Table S1:** Scoring of questions relating to knowledge of ocular genetics and outcomes of gene therapy (Total score: 9)

| **Question** | **Response options** (correct answer(s) are underlined and in bold) | **Scoring** |
| --- | --- | --- |
| Which of the following conditions do you consider to be a monogenic disease (disease traits are controlled by a single gene) | - **Rod-cone dystrophy/retinitis pigmentosa** - Age-related macular degeneration - **Stargardt disease** - Cystoid macular oedema - **Leber Congenital Amaurosis** | 3 points total; +1 point for every correct answer and -1 point for incorrect answer. |
| The **carrier**of a disease gene may be completely healthy | - **Agree** - Neither agree nor disagree - Disagree | +1 point if correct; 0 if incorrect |
| Using current technology, approximately **one in three** ocular genetic tests may come back with an inconclusive result | - **Agree** - Neither agree nor disagree - Disagree | +1 point if correct; 0 if incorrect |
| Gene therapy and stem cell therapy are the **same** treatment^1^ | - Agree - Neither agree nor disagree - **Disagree** | +1 point if correct; 0 if incorrect |
| Patients in Australia have now been treated with approved ocular gene therapy products | - **Agree** - Neither agree nor disagree - Disagree | +1 point if correct; 0 if incorrect |
| The main goal of gene therapy for the eye is to **slow down the disease**^1^ | - **Agree** - Neither agree nor disagree - Disagree | +1 point if correct; 0 if incorrect |
| Having gene therapy for their eye condition means a person **will not pass on an eye condition** to any children they may have in the future^1^ | - Agree - Neither agree nor disagree - **Disagree** | +1 point if correct; 0 if incorrect |

The maximum score was 9. ^1^Questions are adapted from the AGT-Eye tool (Mack et al. *Gene Ther* 2022).^27^

**Supplemental Table S2:** Factors associated with whether optometrists have previously recommended genetic testing for retinal diseases

| Factor | **Univariate analysis** | | **Multivariate analysis** | |
| --- | --- | --- | --- | --- |
|  | OR (95% CI) | p-value | OR (95% CI) | p-value |
| **Age (years)** |  |  |  |  |
| <30 | Ref |  | - |  |
| 30-45 | 1.10 (0.69-1.77) | 0.69 |  |  |
| >45 | 1.33 (0.82-2.19) | 0.25 |  |  |
| **Gender** |  |  |  |  |
| Female or other | Ref |  | - |  |
| Male | 1.00 (0.70-1.44) | 0.99 |  |  |
| **Country** |  |  |  |  |
| Australia | Ref |  | - |  |
| New Zealand | 0.44 (0.16-1.04) | 0.08 |  |  |
| **Region** |  |  |  |  |
| Metropolitan | Ref |  |  |  |
| Regional/Suburban | 1.00 (0.68-1.46) | 0.99 | 1.06 (0.69-1.62) | 0.80 |
| Rural | **2.01 (1.13-3.61)** | **0.018** | **1.97 (1.02-3.81)** | **0.043** |
| **Years in practice** |  |  |  |  |
| ≤10 | Ref |  | - |  |
| 11-20 | 1.02 (0.61-1.70) | 0.93 |  |  |
| >20 | 0.83 (0.56-1.24) | 0.37 |  |  |
| **Practice setting** |  |  |  |  |
| Academic | Ref | - | - |  |
| Corporate | 1.27 (0.44-4.20) | 0.67 |  |  |
| Hospital/public health clinic | 1.71 (0.41-7.63) | 0.47 |  |  |
| Independent | 1.32 (0.47-4.28) | 0.62 |  |  |
| Other | 1.83 (0.37-9.38) | 0.45 |  |  |
| **Hours in practice** |  |  |  |  |
| ≤10 | Ref |  | - |  |
| 11-20 | 0.40 (0.14-1.10) | 0.073 |  |  |
| >20 | 1.21 (0.56-2.79) | 0.63 |  |  |
| **Knowledge score** |  |  |  |  |
| Fail | Ref |  | - |  |
| Pass | 1.53 (1.00-2.40) | 0.057 |  |  |
| **Level of agreement with “If I personally had a genetic eye disease, I would have genetic testing if it was available to me”** |  |  |  |  |
| Strongly agree/Agree | Ref |  | - |  |
| Neither agree nor disagree, disagree, or strongly disagree | 0.63 (0.37-1.02) | 0.068 |  |  |
| **Confidence in explaining the implications of Mendelian inheritance patterns on family planning**^1^ |  |  |  |  |
| No/little confidence | Ref |  | Ref |  |
| Some confidence | 1.49 (0.96-2.32) | 0.078 | 0.75 (0.43-1.29) | 0.31 |
| Comfortable/confident | **1.99 (1.30-3.05)** | **0.002** | 0.68 (0.38-1.21) | 0.20 |
| **Confidence in explaining different reasons for having genetic testing**^1^ |  |  |  |  |
| No/little confidence | Ref |  | Ref |  |
| Some confidence | **3.24 (2.12-4.97)** | **<0.001** | **2.85 (1.72-4.80)** | **<0.001** |
| Comfortable/confident | **4.69 (2.86-7.77)** | **<0.001** | **3.74 (1.95-7.26)** | **<0.001** |
| **Confidence in describing local referral pathways for ocular genetics services**^1^ |  |  |  |  |
| No/little confidence | Ref |  | Ref |  |
| Some confidence | 3.67 (2.34-5.80) | **<0.001** | **2.92 (1.82-4.72)** | **<0.001** |
| Comfortable/confident | 6.97 (4.01-12.5) | **<0.001** | **4.96 (2.75-9.18)** | **<0.001** |

Statistically significant predictors are in bold. **Abbreviations**: OR, Odds Ratio. CI, Confidence Interval.
^1^ Confidence levels were derived from the following responses: No/little confidence: I would not be able to answer questions on this topic or I would not feel comfortable answering questions on this topic; Some confidence: I could answer a few basic questions about this topic; Comfortable/confident: I feel comfortable answering questions about this topic or I feel confident answering questions on this topic.

## Supplemental Table S3: Factors associated with whether optometrists think that they should play a role in initiating genetic testing for patients with retinal disease

| Factor | **Univariate analysis** | | **Multivariate analysis** | |
| --- | --- | --- | --- | --- |
|  | OR (95% CI) | p-value | OR (95% CI) | p-value |
| **Age (years)** |  |  |  |  |
| <30 | Ref |  | Ref |  |
| 30-45 | **0.59 (0.36–0.96)** | **0.034** | **0.53 (0.31-0.89)** | **0.018** |
| >45 | 0.64 (0.40-1.02) | 0.064 | 0.67 (0.41-1.10) | 0.12 |
| **Gender** |  |  |  |  |
| Female or other | Ref |  | - |  |
| Male | 0.82 (0.57-1.16) | 0.26 |  |  |
| **Country** |  |  |  |  |
| Australia | Ref |  | - |  |
| New Zealand | 0.69 (0.32-1.49) | 0.34 |  |  |
| **Region** |  |  |  |  |
| Metropolitan | Ref |  |  |  |
| Regional/Suburban | 0.91 (0.63–1.33) | 0.63 | - |  |
| Rural | 1.09 (0.61-1.98) | 0.78 |  |  |
| **Years in practice** |  |  |  |  |
| ≤10 | Ref |  | - |  |
| 11-20 | 0.87 (0.52-1.46) | 0.60 |  |  |
| >20 | 0.69 (0.46-1.02) | 0.062 |  |  |
| **Practice setting** |  |  |  |  |
| Academic | Ref |  | - |  |
| Corporate | 0.40 (0.11-1.20) | 0.12 |  |  |
| Hospital/public health clinic | 0.43 (0.09-1.87) | 0.27 |  |  |
| Independent | 0.49 (0.13-1.44) | 0.23 |  |  |
| Other | 1.50 (0.24-12.6) | 0.68 |  |  |
| **Hours in practice** |  |  |  |  |
| ≤10 | Ref |  | - |  |
| 11-20 | 0.93 (0.37-2.31) | 0.88 |  |  |
| >20 | 0.91 (0.41-1.98) | 0.82 |  |  |
| **Knowledge score** |  |  |  |  |
| Fail | Ref |  | - |  |
| Pass | 1.27 (0.84-1.92) | 0.25 |  |  |
| **Level of agreement with “If I personally had a genetic eye disease, I would have genetic testing if it was available to me”** |  |  |  |  |
| Strongly agree/Agree | Ref |  | Ref |  |
| Neither agree nor disagree, disagree, or strongly disagree | **0.38 (0.24–0.61)** | **<0.001** | **0.42 (0.26-0.69)** | **<0.001** |
| **Confidence in explaining the implications of Mendelian inheritance patterns on family planning^1^** |  |  |  |  |
| No/little confidence | Ref |  | Ref |  |
| Some confidence | 1.13 (0.74–1.73) | 0.58 | 0.79 (0.48-1.30) | 0.36 |
| Comfortable/confident | **2.13 (1.38-3.32)** | **<0.001** | 1.22 (0.70-2.13) | 0.48 |
| **Confidence in explaining different reasons for having genetic testing^1^** |  |  |  |  |
| No/little confidence | Ref |  | Ref |  |
| Some confidence | **1.71 (1.14-2.56)** | **0.010** | **1.67 (1.04-2.70)** | **0.036** |
| Comfortable/confident | **4.08 (2.37-7.27)** | **<0.001** | **3.01 (1.54-6.05)** | **0.002** |
| **Confidence in describing local referral pathways for ocular genetics services^1^** |  |  |  |  |
| No/little confidence | Ref |  | Ref |  |
| Some confidence | 1.90 (1.21-3.03) | 0.006 | 1.40 (0.86-2.29) | 0.18 |
| Comfortable/confident | 2.21 (1.28-3.94) | 0.006 | 1.58 (0.87-2.96) | 0.14 |

Statistically significant predictors are in bold. **Abbreviations**: OR, Odds Ratio. CI, Confidence Interval.
^1^ Confidence levels were derived from the following responses: No/little confidence: I would not be able to answer questions on this topic or I would not feel comfortable answering questions on this topic; Some confidence: I could answer a few basic questions about this topic; Comfortable/confident: I feel comfortable answering questions about this topic or I feel confident answering questions on this topic.
